# Editorial: Dietary intake, eating behavior and health outcomes

**DOI:** 10.3389/fnut.2023.1158970

**Published:** 2023-02-24

**Authors:** Rafaela Rosário, Tuyen V. Duong, Inês Fronteira

**Affiliations:** ^1^School of Nursing, University of Minho, Braga, Portugal; ^2^Health Sciences Research Unit: Nursing (UICISA: E), Nursing School of Coimbra (ESEnfC), Coimbra, Portugal; ^3^Nursing Research Centre, University of Minho, Braga, Portugal; ^4^School of Nutrition and Health Sciences, Taipei Medical University, Taipei, Taiwan; ^5^Global Health and Tropical Medicine, Instituto de Higiene e Medicina Tropical – Universidade NOVA de Lisboa, Lisbon, Portugal

**Keywords:** dietary intake, eating behavior, health outcomes, non-communicable disease, nutrition

Dietary intake and eating behaviors are important determinants of non-communicable diseases (NCDs) and have been widely investigated (1). The association between dietary intakes (e.g., fruits, vegetables, processed meat, and trans-fat) and NCDs (e.g., obesity, cardiovascular diseases (CVD), diabetes, and cancer) have been described. The associations varied by socioeconomic and the burden differed by demographic conditions (2, 3). Nevertheless, due to the complexity of measuring exposures related to dietary intake, the evidence base is mainly observational and lacking experimental designs. Additionally, there is scant evidence on the effectiveness of health promotion strategies focusing dietary consumption and behavior, on health outcomes.

Bring in mind the complexity of studying dietary intake and its effects in health, the Frontiers in Nutrition dedicated a specific topic to *Dietary intake, eating behavior and health outcomes*. A total of 105 studies were submitted with 35 being selected for publication after peer-review.

In this editorial, we focus on those 35 studies addressing dietary and nutrition intake [e.g., fatty acids (Fan et al.; Tan and Shin), pro-inflammatory diet (Ruan et al.; Schütte et al.; Wang Q. et al.; Yan et al.; Zhao et al.), fruit and vegetables (Jin et al.; Wang R. et al.), meat (Chen et al.; Wu et al.), and others (AL-Mohaithef; Buso et al.; Jiang et al.; Li et al.; Lei et al.; Liu et al.; Sousa et al.; Yu et al.)], dietary patterns and behaviors (Bai et al.; Cui et al.; D'Esposito et al.; Di Maso et al.; Kim and Kim; Nguyen et al.; Palomar-Croset al.; Park; Zhang et al.) and dietary diversity (Hu et al.; Kim and Kim; Qu et al.; Zhou et al.). Also, two studies focused on socioeconomic disadvantage (Isaura et al.) and on the neighborhood effects on eating behaviors among elders (Liu and Yu).

In the study of Qu et al. the dietary diversity score was associated with a reduction in the risk of mortality. Hu et al. in their study with preschool children from poor ethnic minority areas found significant associations between dietary diversity, nutrient adequacy, and anthropometric status. Also Isaura et al. comprehensively discussed how childhood and early socioeconomic disadvantage is related to adult food security status and poor health in children. Focusing on older adults from China, the investigation of Liu and Yu about the effects of neighborhood diet quality on the eating behaviors of older adults living in the same community is sound. The neighborhood effects were manifested in increased consumption of vegetables and fruits, meat, eggs, and dairy products.

The studies from this Research Topic addressed several health outcomes, namely diabetes (Yu et al.; Zhao et al.), cancer (Fan et al.; Jiang et al.; Jin et al.; Li et al.; Palomar-Cros et al.; Park; Wang R. et al.; Wu et al.), obesity and weight status (AL-Mohaithef; Buso et al.; Hu et al.; Kim and Kim; Lei et al.; Zhou et al.), CVD (Wang Q. et al.; Xie et al.; Zhang et al.), among others (Cui et al.; D'Esposito et al.; Di Maso et al.; Liu et al.; Ruan et al.; Schütte et al.; Tan and Shin; Yan et al.), including psychological issues (Berbegal et al.; Nguyen et al.; Sousa et al.).

In the Yu et al.'s study, intakes of branched-chain amino acids was associated with higher risk of type 2 diabetes, while Zhao et al., presented positive association between dietary acid load during early pregnancy and the risk of gestational diabetes mellitus.

In an updated systematic review and meta-analysis, Wang R. et al. found positive associations between dietary acid load and cancer risk and prognosis. Fan et al. found significant differences between dietary fatty acids intake in patients with oral cancer and controls. A positive association between the saturated fatty acids pattern and risk of oral cancer was observed, even after adjusting for potential confounders. Also beholding fatty acid intake, Tan and Shin discussed the preventative benefit of consuming oily fish and its fatty acid intake on non-alcoholic fatty liver disease, notably in South Korean women. In a large U.S. prospective cohort, Jiang et al. indicated that a moderate consumption of carrots was associated with a lower colorectal cancer incidence. In their Mendelian randomization study, Li et al., described direct associations between coffee and caffeine consumption and renal cell carcinoma, although suggesting the need to conduct further studies on the matter. Intake of dried fruit was considered protective on some site-specific cancers in the study of Jin et al.. The authors emphasized health education and an adjustment of dietary proportion for primary prevention of cancer. Regarding vegetable consumption, Park found inverse associations between their consumption and colon cancer. In the same study, the author reported positive associations between red meat and colon cancer and mortality. Also Wu et al. suggested that intake of processed meat was associated with an increased risk of lung cancer. In their study, Palomar-Cros et al. found significant associations between breakfast time and breast cancer.

Concerning weight status, Buso et al. suggested positive associations between consumption of both sugar-sweetened beverages and low/no-calorie beverages and weight-related outcomes. Dietary diversity was associated with body mass index in youth in the study of Zhou et al., while in the study of Hu et al. in preschool children, dietary diversity was associated with nutrient adequacy and other health outcomes. In addition, Kim and Kim suggested that psychosocial stress contributed to abdominal obesity by interacting with a low dietary variety score. Low carbohydrate diets and low fat diets had significant effects on metabolic risk factors and weight loss in the study of Lei et al. in adults with overweight and obesity, although the long-term effects of various sources of carbohydrates or fat in both diets need to be further studied. AL-Mohaithef addressed that vegan and vegetarian diets have increased in Saudi adults and those with a vegetarian diet showed a better lifestyle (e.g., higher physical activity level, higher consumption of fruits, vegetables, dairy products), low intake of fast-foods and fizzy beverages and was significantly associated with body mass index.

Concerning CVD, Zhang et al. reported prospective inverse associations between adherence to the 2015-2020 Dietary Guidelines for Americans and CVD risk. Also, Wang Q. et al., found inverse associations between urinary thiocyanate, a candidate biomarker of cruciferous vegetable intake, and CVD and total mortality among non-smoking adults. When studying people with diabetes, Xie et al. found that higher eating frequency was independently related to lower all-cause and CVDs-related mortality.

Anxiety was addressed in the study of Sousa et al. suggesting that the consumption of fermented dairy products had a positive effect on reducing anxiety in young university students. Also, Nguyen et al. found that during the pandemic, fear of COVID-19 and cigarette smoking had adverse impacts on medical students' psychological health. The results suggested that staying physically active and having healthy eating behaviors could potentially protect medical students from anxiety and depressive symptoms. Finally, Berbegal et al. provided further insights about the importance of adiposity in health and memory function, reporting that some measures of adiposity (e.g., weight, BMI, waist-hip ratio index) were inversely associated with memory function.

In their study, Schütte et al. found inverse associations between a pro-inflammatory dietary pattern and atopic outcome in children. In the same study, it was emphasized that this pattern reduced the buffering capacity of the individual against harmful environmental exposures or triggers. Also, D'Esposito et al. showed that age, body mass index, and lifestyles were associated with specific cytokines, potential markers for low-grade chronic inflammation.

In their study, Yan et al. confirmed the hypothesis that proinflammatory diets contribute to increase all-cause mortality in adults with chronic kidney disease. Concerning breastfeeding mothers, Di Maso et al., on behalf of MEDIDIET Working Group Members, found that a high adherence to the Mediterranean diet was associated with human milk composition, namely the milk's content of specific fatty acids.

In their study, Liu et al. confirmed a dose-response association between higher alcohol consumption and inflammatory bowel disease risk. Concerning other health outcomes, Chen et al. established associations between red meat, remarkably beef intake, and risk of rheumatoid arthritis. Ruan et al. when studying the dietary inflammatory potential, as estimated by the dietary inflammation index score, found that it is positively associated with erectile dysfunction among US males. Bai et al. focusing on some lifestyles determinants such as cigarette smoking, alcohol abuse, and decaffeinated coffee are associated with gallstone disease, whereas tea consumption can decrease the risk of gallstones due to the effect of caffeine metabolism on polyphenol intake. Interestingly, Cui et al. identified three dietary patterns in their study (i.e., “high protein pattern,” “snack food pattern,” and “vegetarian food pattern”), all of them showing a high linear association with high-altitude polycythemia.

## Author contributions

RR formulated a draft. IF and TD revised the manuscript. All authors contributed to the article and approved the submitted version.
